# Hypercalcemia in Lung Cancer due to Simultaneously Elevated PTHrP and Ectopic Calcitriol Production: First Case Report

**DOI:** 10.1155/2017/2583217

**Published:** 2017-07-02

**Authors:** Saed Nemr, Sunitha Alluri, Dhivya Sundaramurthy, Daniel Landry, Gregory Braden

**Affiliations:** ^1^Nephrology Division, Baystate Medical Center, Tufts University School of Medicine, Springfield, MA, USA; ^2^Division of Hospital Medicine, Baystate Medical Center, Tufts University School of Medicine, Springfield, MA, USA

## Abstract

Calcitriol-mediated hypercalcemia has been reported in malignant lymphomas and granulomatous diseases but not in lung carcinoma. We describe a patient with squamous cell lung carcinoma with hypercalcemia and elevated calcitriol levels. A 60-year-old Caucasian male patient with stage IIIB squamous cell lung cancer developed hypercalcemia at 14.8 mg/dL two years after receiving chemotherapy and radiotherapy where labs showed a serum intact PTH: 7 pg/mL, PTHrP: 30 pmol/L, 1,25-hydroxyvitamin D (calcitriol): 76 pg/mL, and 25-hydroxyvitamin D levels: <4 ng/mL. Calcitriol levels were elevated despite undetectable 25-hydroxyvitamin D levels. There are no reported lung cancer cases with elevated calcitriol as an etiology of hypercalcemia. We believe that the elevated calcitriol levels in this case were due to a PTHrP-independent mechanism, possibly from either ectopic production of calcitriol in tumor cells or from increased activity of 1-alpha hydroxylase in the same cells. The patient died before the effects of prednisone therapy could be assessed. Studies are needed to investigate the cellular source of calcitriol and its role in hypercalcemia in patients with lung cancer.

## 1. Introduction

Calcitriol-mediated hypercalcemia has been reported in malignant lymphomas, both Hodgkin's and non-Hodgkin's types [1–5], and diseases involving granulomatous pathology such as sarcoidosis and tuberculosis [6, 7], but not in lung carcinoma. Dysregulated calcitriol production is a very rare occurrence in patients with hypercalcemia associated with solid tumors, where suppressed serum calcitriol levels are characteristic. However, to date, lung carcinoma has not been associated with hypercalcemia from ectopic calcitriol production. We report here one exceptional case of a patient with squamous cell lung carcinoma with hypercalcemia and elevated PTHrP and calcitriol levels.

## 2. Case Presentation

A 60-year-old Caucasian male with stage IIIB squamous cell lung cancer developed hypercalcemia 2 years after radiotherapy and chemotherapy with two cycles of carboplatin and paclitaxel which initially reduced the size of the lung mass and mediastinal lymphadenopathy. He had lung cancer progression and thereafter he received vinorelbine without response. His calcium level rose to 13.6 mg/dL with a normal serum creatinine of 0.7 mg/dl. He did not have any signs or symptoms of hypercalcemia. Hypercalcemia workup was not done at that time. He responded to intravenous 0.9% normal saline and 4 mg of zoledronic acid with rapid improvement in calcium levels and he was discharged on calcitonin nasal spray. He was readmitted with septic shock and his calcium levels were noted to be low; hence, calcitonin was discontinued. A month later, he was admitted for shortness of breath and his calcium level was 14.8 mg/dL. Physical exam at this time revealed decreased skin turgor, clear lungs, a cardiac flow murmur, and no leg edema. Calcitonin was resumed and he was started on intravenous 0.9% normal saline which produced minimal improvement in hypercalcemia. Treatment with zoledronic acid 3 mg and furosemide was initiated but the calcium levels remained between 12 and 13 mg/dL. Workup now revealed the following: serum intact PTH: 7 pg/mL (15–65 pg/mL), PTHrP: 30 pmol/L (<2.0 pmol/L), 1,25-hydroxyvitamin D (calcitriol): 76 pg/ml (18–64 pg/mL), 25-hydroxyvitamin D levels: <4 ng/mL (20–50 ng/mL), and phosphorus level: 1.8 mg/dl (2.5–4.5 mg/dL). Hypercalcemia workup was repeated and confirmed the previous results: serum intact PTH: 7 pg/mL (15–65 pg/mL), PTHrP: 38 pmol/L (<2.0 pmol/L), 1,25-hydroxyvitamin D (calcitriol): 71 pg/ml (18–64 pg/mL), 25-hydroxyvitamin D levels: <4 ng/mL (20–50 ng/mL). Sadly the patient opted for comfort measures and active management plans were terminated. Calcitriol levels were elevated despite undetectable 25-hydroxyvitamin D levels.

## 3. Discussion

Malignancy related hypercalcemia can be broadly divided into three categories: (1) humoral hypercalcemia of malignancy which is PTHrP mediated, (2) local osteolytic hypercalcemia, and (3) dysregulated calcitriol production [4]. See Table 1.

In this case of squamous cell lung cancer, calcitriol levels were elevated, and serum PTH was suppressed; however, the serum PTHrP levels were also elevated above 30 pmol/L. The normal serum concentration of PTHrP in the blood is less than 2.0 pmol/L; most hypercalcemic patients with solid organ malignancies have PTHrP levels higher than this value [8–10]. In addition, the elevated serum calcium was refractory to standard therapy including IV 0.9% normal saline, calcitonin, and zoledronic acid prompting a search for additional hypercalcemic factors. It is possible that the hypercalcemia in our case is due to two mechanisms mediated by both ectopic calcitriol and PTHrP production. Hypercalcemia secondary to dual mechanisms has been reported in hematological malignancies and certain solid tumors like ovarian cancers, pancreatic neuroendocrine tumors, seminomas, and renal cell carcinomas [11, 12]. There are no reported cases of hypercalcemia in squamous cell lung carcinoma associated with elevated calcitriol levels and PTHrP at the same time.

Extrarenal expression of 1-alpha hydroxylase occurs in alveolar macrophages, the placenta, keratinocytes, prostate cancer, colon cancer, breast cancer, pancreas, brain, adrenal medulla, and vascular endothelial cells. Unfortunately in our case, the original lung biopsy was done long before the patient developed hypercalcemia, and staining the biopsy for 1,25-dihydroxyvitamin D to confirm ectopic production of calcitriol or increased activity of 1 alpha hydroxylase in tumor cells was not possible. Although PTHrP does increase 1-alpha hydroxylase activity and calcitriol production in mice models, it does not increase calcitriol production in humans [13, 14]. We believe that the elevated calcitriol levels in this case were due to a PTHrP-independent mechanism, possibly from either ectopic production of calcitriol in tumor cells or increased activity of 1-alpha hydroxylase in the same cells. According to Zehnder et al. [15], there are important differences in regulation of renal and extrarenal calcitriol production. Extrarenal synthesis of calcitriol is very much dependent on availability of its substrate 25-hydroxyvitamin D. In our case, the 25-hydroxyvitamin D levels were very low which makes ectopic calcitriol production more likely.

The cellular source for increased calcitriol production in lung cancer might be from either lung cancer cells or alveolar macrophages or vascular endothelial cells (possible neovascularization in the setting of cancer). Increased expression of 1-alpha hydroxylase in alveolar macrophages associated with lung cancer is possible. However, in patients with cancer, 1-alpha-hydroxylase-mRNA levels correlate well with serum 1-alpha,25-dihydroxyvitamin D3 concentration and the ratio of 1-alpha,25-dihydroxyvitamin D3 to 25-hydroxyvitamin D3 but not with calcium metabolism [16]. Also, enzyme expression increased depending on the clinical stage of lung cancer. In our case, hypercalcemia was noted late in the course of disease which also indicated high tumor burden and a poor prognosis.

The usual modalities of treatment of PTHrP-mediated humoral hypercalcemia of malignancy, including intravenous 0.9% normal saline, intravenous bisphosphonates, calcitonin, and loop diuretics were minimally effective in this case. Corticosteroids are the first-line agents for treatment of calcitriol-mediated hypercalcemia. Unfortunately, our patient died before prednisone therapy could be assessed.

It will be worthwhile to conduct studies to investigate the cellular source of calcitriol and its role in hypercalcemia in patients with lung cancer.

## 4. Conclusion

We believe that the refractory hypercalcemia in this patient was due to elevated calcitriol levels due to a PTHrP-independent mechanism, possibly from either ectopic production of calcitriol in tumor cells or from increased activity of 1-alpha hydroxylase in the same cells or alveolar macrophages. Calcitriol levels should be measured in hypercalcemia lung cancer patients to detect ectopic calcitriol production which could be treated with prednisone.

## Figures and Tables

**Table 1:** Hypercalcemia of malignancy.

Tests	Calcitriol-mediated	Humoral hypercalcemia	Local osteolytic	Our patient
Serum PTH	Decreased	Decreased	Decreased	Decreased
Serum PTHrP	Undetectable	Increased	Undetectable	Increased
1,25-dihydroxyvitamin D	Increased	Decreased	Decreased	Increased
Phosphate	Normal/increased	Decreased	Normal	Decreased

## Data Access

All data used in the case report are stored in the Cerner Electronic Medical Record of Baystate Medical Center.
